# Activation of focal adhesion kinase enhances the adhesion and invasion of pancreatic cancer cells via extracellular signal-regulated kinase-1/2 signaling pathway activation

**DOI:** 10.1186/1476-4598-4-37

**Published:** 2005-10-06

**Authors:** Hirozumi Sawai, Yuji Okada, Hitoshi Funahashi, Yoichi Matsuo, Hiroki Takahashi, Hiromitsu Takeyama, Tadao Manabe

**Affiliations:** 1 Department of Gastroenterological Surgery, Nagoya City University Graduate School of Medical Sciences, Nagoya 4678601, Japan

## Abstract

**Background:**

Interaction with integrin and focal adhesion kinase (FAK) regulates the cancer cell adhesion and invasion into extracellular matrix (ECM). In addition, phosphorylation of FAK correlates with the increase of cell motility and invasion. Adhesion and spreading of cancer cells on a variety of ECM proteins, including collagen type IV (Coll IV), leads to an increase in tyrosine phosphorylation and activation of FAK. In this study, we investigated the mechanism of activation of FAK and its downstream extracellular signal-regulated kinase (ERK)-1/2 signaling following stimulation by interleukin (IL)-1α and adhesion to ECM with subsequent enhancement of pancreatic cancer cell adhesion and invasion.

**Results:**

In immunoblotting analysis, all three pancreatic cancer cell lines (AsPC-1, BxPC-3, and Capan-2) expressed the protein of FAK and β_1 _integrin. Enhancement of FAK protein association with β_1 _integrin when cells were plated on Coll IV was more increased by stimulation with IL-1α. Preincubation with anti-β_1 _integrin antibody and FAK siRNA transfection inhibited the association of FAK with β_1 _integrin of pancreatic cancer cells. FAK phosphorylation was observed by adhesion to Coll IV, furthermore, stronger FAK phosphorylation was observed by stimulation with IL-1α of pancreatic cancer cells adhered to Coll IV in time-dependent manner. Genistein, a tyrosine kinase inhibitor, markedly inhibited the FAK phosphorylation. IL-1α stimulation and Coll IV adhesion enhanced the activation of Ras, as evidenced by the increased Ras-GTP levels in pancreatic cancer cells. Activation of Ras correlated with the phosphorylation of ERK. While not statistical affecting the apoptosis of pancreatic cancer cells, IL-1α-induced adhesion and invasion on Coll IV were inhibited with FAK gene silencing by siRNA, β_1 _integrin blocking, and inhibition of FAK phosphorylation. PD98059, a MEK inhibitor, also inhibited IL-1α-induced enhancement of adhesion and invasion in pancreatic cancer cells.

**Conclusion:**

Our results demonstrated that activation of FAK is involved with the aggressive capability in pancreatic cancer through Ras/ERK signaling pathway. Based on our results, we suggest that the modification of IL-1, FAK, and integrins functions might be a novel therapeutic approach to aggressive spread of pancreatic cancer.

## Background

Integrin binding to extracellular matrix (ECM) protein or integrin crosslinking increases tyrosine phosphorylation of focal adhesion kinase (FAK) [1,2]. FAK is a tyrosine kinase considered a central molecule in integrin-mediated signaling, and it is involved in cellular motility and protection against apoptosis [3-7]. The carboxyl-terminal tyrosine residue (tyr^397^) of FAK, constitutes a major site of phosphorylation, appears important for the tyrosine phosphorylation of focal complex associated proteins, and creates a high-affinity binding site recognized by the SH-2 domain of the Src family [8,9]. In vitro, the N-terminal domain of FAK binds directly to peptides corresponding to the cytoplasmic domain of integrin β subunits [2,10]. In addition, overexpression and phosphorylation of FAK correlates with the increase of cell motility and invasion [4,5,11,12]. Adhesion and spreading of cells on a variety of ECM proteins, including collagen type IV (Coll IV), leads to an increase in tyrosine phosphorylation and activation of FAK [3,4,7]. Furthermore, suppression of adhesion induced tyrosine phosphorylation of FAK may interrupt cancer cell-ECM interactions and affect the invasive and metastatic potential of cancer cells. Based on these results, considerable evidence points to a critical role of FAK participating in cancer cell-ECM interactions.

The integrin family ECM receptors are key mediators of cell proliferation and cell survival. Integrin-mediated cell adhesion is required for cell motility and also affects cell proliferation and invasion in many systems [13-15]. We previously proved that enhancement of α_6_β_1_-integrin expression by interleukin (IL)-1α acting through IL-1 receptor type I (IL-1RI) plays an important role in metastatic and invasive behaviors of pancreatic cancer, and that strong expression of α_6 _integrin in cancerous tissues significantly correlated with poor prognosis of pancreatic cancer patients [15,16]. β_1 _integrin is also reported to play an important role in invasiveness and metastasis formation of cancer cells [17-19].

Integrin-ECM interactions also activate signaling cascades such as extracellular signal-regulated kinase-1/2 (ERK1/2), mitogen activated protein kinase (MAPK), phosphatidylinositol 3-kinase (PI3-K), and Akt [2-5,20-23]. Especially, the downstream targets of Ras signaling pathway are ERK1/2, which have been found to be regulated by activation of FAK with respect to different matrix components [3,21,24]. Therefore, integrin binding to the ECM creates and activates a bipartite kinase complex and transduces external stimuli from the ECM to the nucleus.

In this study, we investigated the mechanism of activation of FAK and its downstream ERK1/2 signaling following adhesion to ECM. Our results suggest that activation of FAK enhances the adhesive and invasive capabilities of pancreatic cancer cells through Ras/ERK signaling pathway.

## Results

### Expression of FAK and β_1 _integrin in pancreatic cancer cells

**Figure 1:**
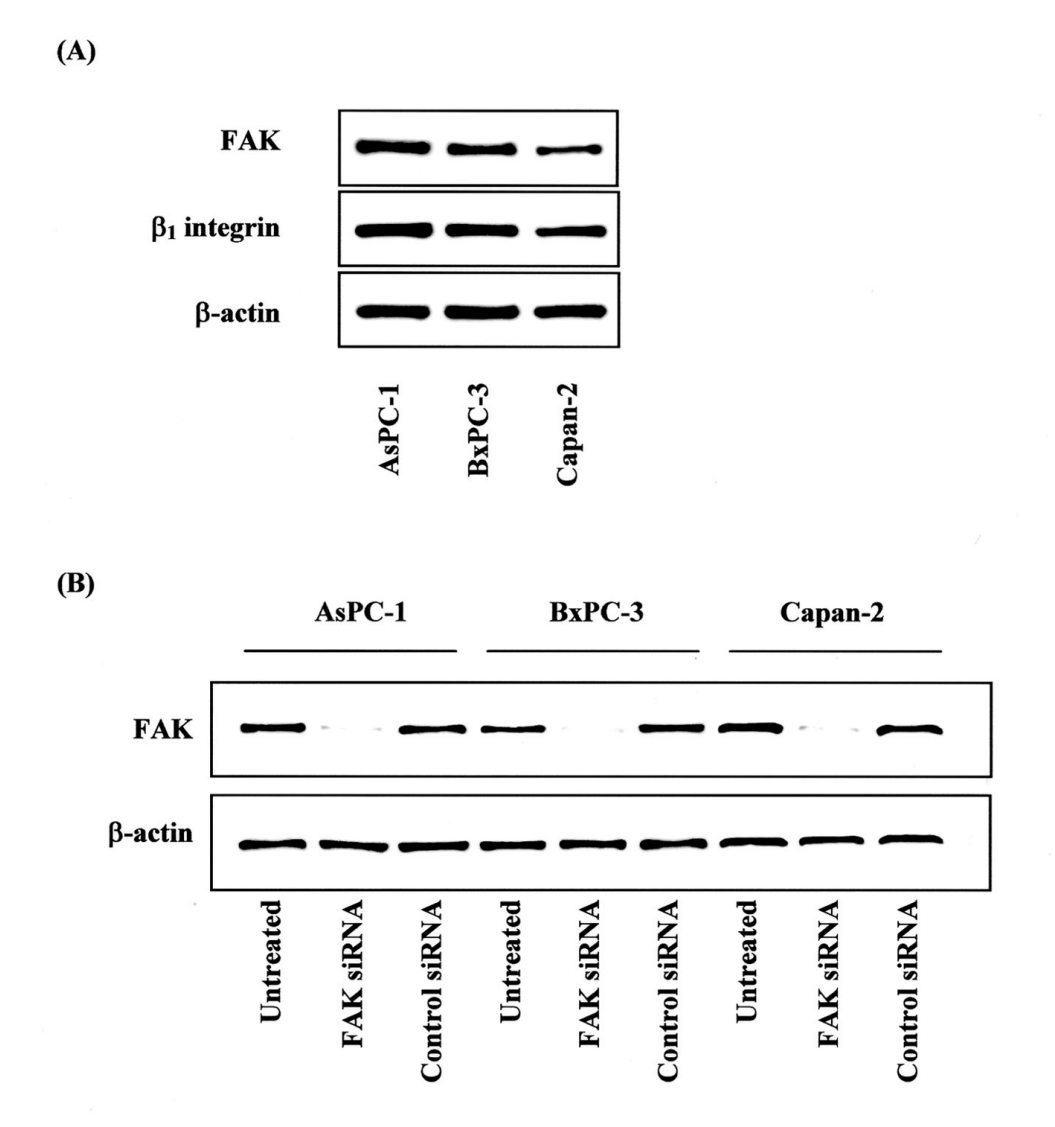
Expression of FAK and β_1 _integrin in pancreatic cancer cells. **(A) **FAK and β_1 _integrin protein expression in pancreatic cancer cell lines was determined in whole cell lysates by Western blotting analysis. Fifty micrograms of total cell lysates was separated on 10 % SDS-PAGE and transferred to polyvinylidene difluoride membranes. Membranes were probed with antibodies against FAK and β_1 _integrin. The β-actin Western blot served as a loading control. **(B) **Knockdown of FAK expression by siRNA was confirmed by immunoblotting in all three pancreatic cancer cells. Re-probing with an anti-β-actin antibody served as a control.

In immunoblotting analysis, all three pancreatic cancer cell lines also expressed FAK and β_1 _integrin (Fig. 1A). In this study, we transfected all three pancreatic cancer cells with siRNA that specifically targets FAK. Downregulation of FAK protein expression by siRNA was confirmed by immunoblotting. Transfection of siRNA resulted in a near total loss of FAK expression (Fig. 1B).

### Interaction between FAK and β_1 _integrin

**Figure 2:**
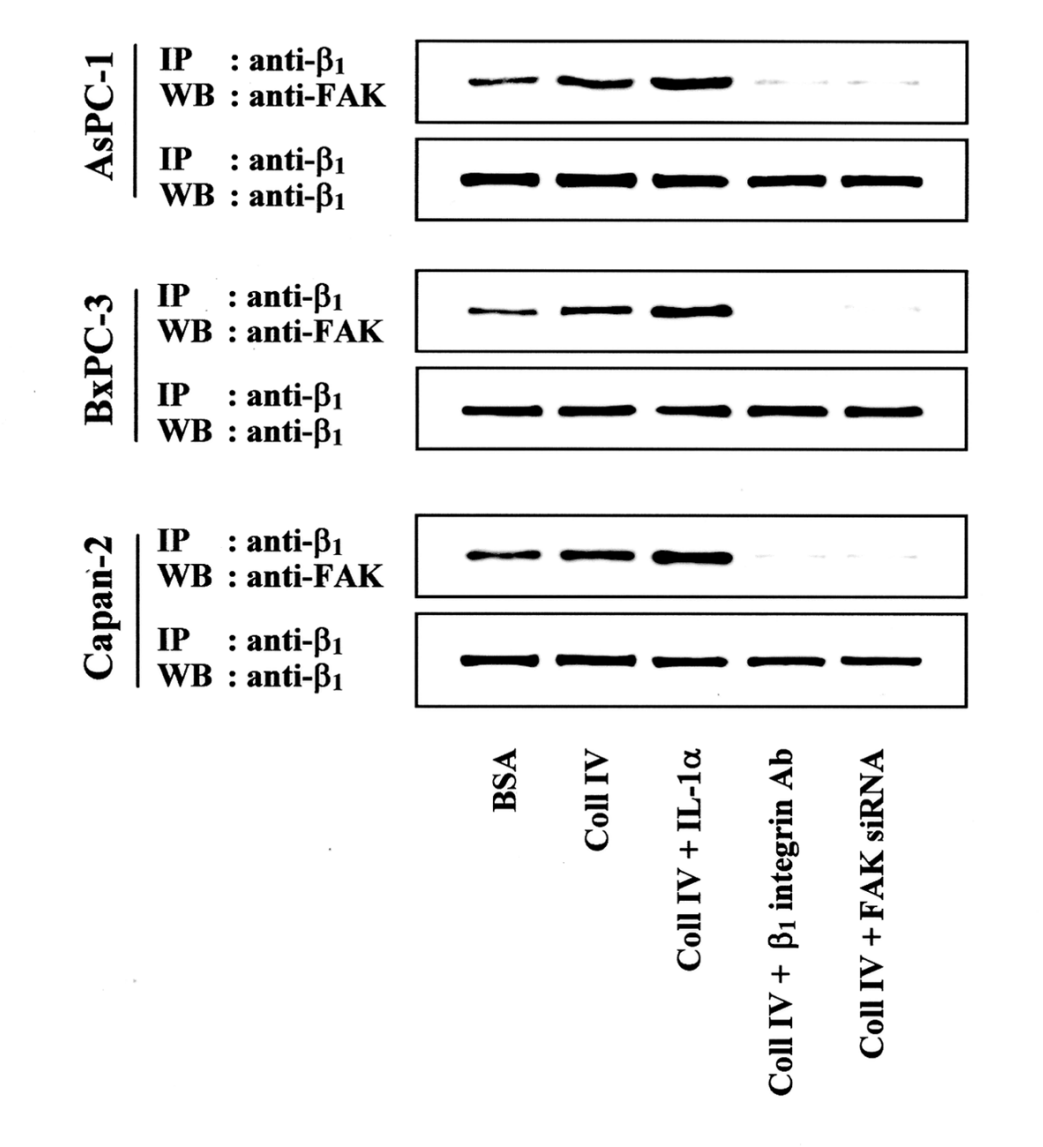
FAK and β_1 _integrin subunit interaction in AsPC-1, BxPC-3, and Capan-2 cells. After incubating on Coll IV with 10 ng/ml IL-1α or 0.5 μg/ml anti-β_1 _integrin antibodies for 24 h, cells were added (2 × 10^5^cells/well) to each well and incubated at 37°C and 5% CO_2 _for 30 min. After removing unattached cells, total cell lysates were immunoprecipitated with antibodies against β_1 _integrin subunit. Samples were resolved in 10 % SDS-PAGE gel under nonreducing conditions and transferred to polyvinylidene difluoride membranes. Membranes were then probed with anti-FAK or anti-β_1 _integrin antibodies. Samples from cells incubated on 3 % BSA were served as control.

In order to determine if FAK interacts with β_1 _integrin subunit, β_1 _integrin was immunoprecipitated from cell lysates of AsPC-1, BxPC-3, and Capan-2 cells and Western blotted using anti-FAK antibody. As shown Fig. 2, more FAK protein associated with β_1 _integrin when cells were plated on Coll IV. Stimulation with recombinant human IL-1α (rIL-1α) of pancreatic cancer cells on Coll IV furthermore enhanced the association of FAK with β_1 _integrin. Preincubation with anti-β_1 _integrin antibody and FAK siRNA inhibited the association of FAK with β_1 _integrin of pancreatic cancer cells (Fig. 2).

### Phosphorylation of FAK was enhanced by Coll IV adhesion and IL-1α stimulation

**Figure 3:**
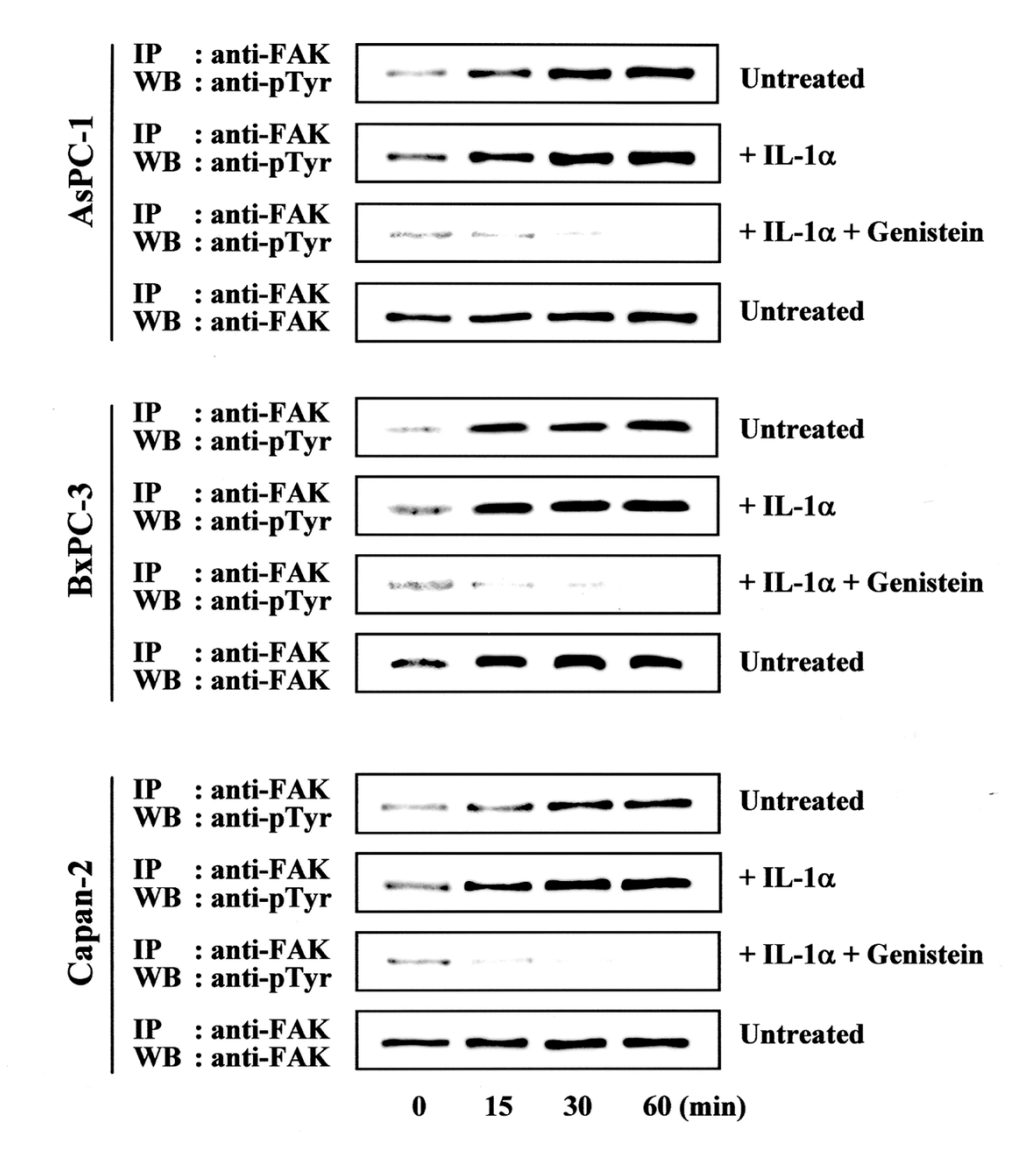
Phosphorylation of FAK in AsPC-1, BxPC-3, and Capan-2 cells. After incubating on Coll IV with 10 ng/ml IL-1α and/or 60 μM Genistein for 24 h, cells were added (2 × 10^5 ^cells/well) to each well and incubated at 37°C and 5 % CO_2 _for 15, 30, or 60 min. After removing unattached cells, cells were collected from each time point and lysed by lysis buffer and immunoprecipated with FAK antibody as described in Methods and materials. Effect of IL-1α and Genistein on Coll IV mediated phosphorylation of FAK was demonstrated. IP, immunoprecipitation; WB, Western blot.

FAK activity was examined by FAK phosphorylation and total tyrosine phosphorylation in AsPC-1, BxPC-3, and Capan-2 cells. The lysates of pancreatic cancer cells were analyzed by immunoprecipitation with antibodies to FAK followed by Western blotting with an antibody which is specific for anti-phosphotyrosine (4G10). FAK activation was observed within 15 min of adhesion to Coll IV and remained high for 60 min. (Fig. 3). Stronger FAK phosphorylation was observed by stimulation with rIL-1α of pancreatic cancer cells adhered to Coll IV in time-dependent manner (Fig. 3). In contrast, incubation with Genistein, a tyrosine kinase inhibitor, markedly inhibited the FAK phosphorylation.

#### Effect of FAK gene silencing and β_1 _integrin blocking on apoptosis of pancreatic cancer cells

After treated/untreated with FAK siRNA or control siRNA, pancreatic cancer cells were incubated with/without anti-β_1 _integrin antibody for 24 h, and then terminal deoxynucleotidyl transferase-mediated nick end labeling (TUNEL) assay was performed to investigate whether knockdown of FAK expression and β_1 _integrin blocking had any effects on pancreatic cancer apoptosis. Both FAK siRNA transfection and incubation with β_1 _integrin antibody induced a slight increase in the apoptotic fraction of calls in normal culture conditions, however, there was no statistical difference among these treatments (Table 1).

**Table 1:** Effect of FAK siRNA and β_1 _integrin blocking on apoptosis of pancreatic cancer cells

	Apoptotic fraction (%)			
	
Cell line	Untreated	Anti-β_1 _integrin antibody	FAK siRNA	Control siRNA
AsPC-1	1.37 ± 0.33	1.89 ± 0.51	2.20 ± 0.66	1.44 ± 0.54
BxPC-3	1.99 ± 0.31	2.38 ± 0.51	2.56 ± 0.53	2.02 ± 0.4
Capan-2	2.44 ± 0.30	2.59 ± 0.37	3.01 ± 0.52	2.50 ± 0.43

AsPC-1, BxPC-3, and Capan-2 cells were treated with anti-β_1 _integrin antibody, FAK siRNA, or control siRNA and incubated for 24 h, and then detection of apoptosis was performed by TUNEL assay and flow cytometry. Statistical significance was tested by one-way analysis of variance and post hoc test (Turkey Kramer multiple comparisons). All data are expressed as mean ± s.d. There was no significant difference among these treatments.

### Involvement of FAK with adhesive and invasive capabilities of pancreatic cancer cells

**Figure 4:**
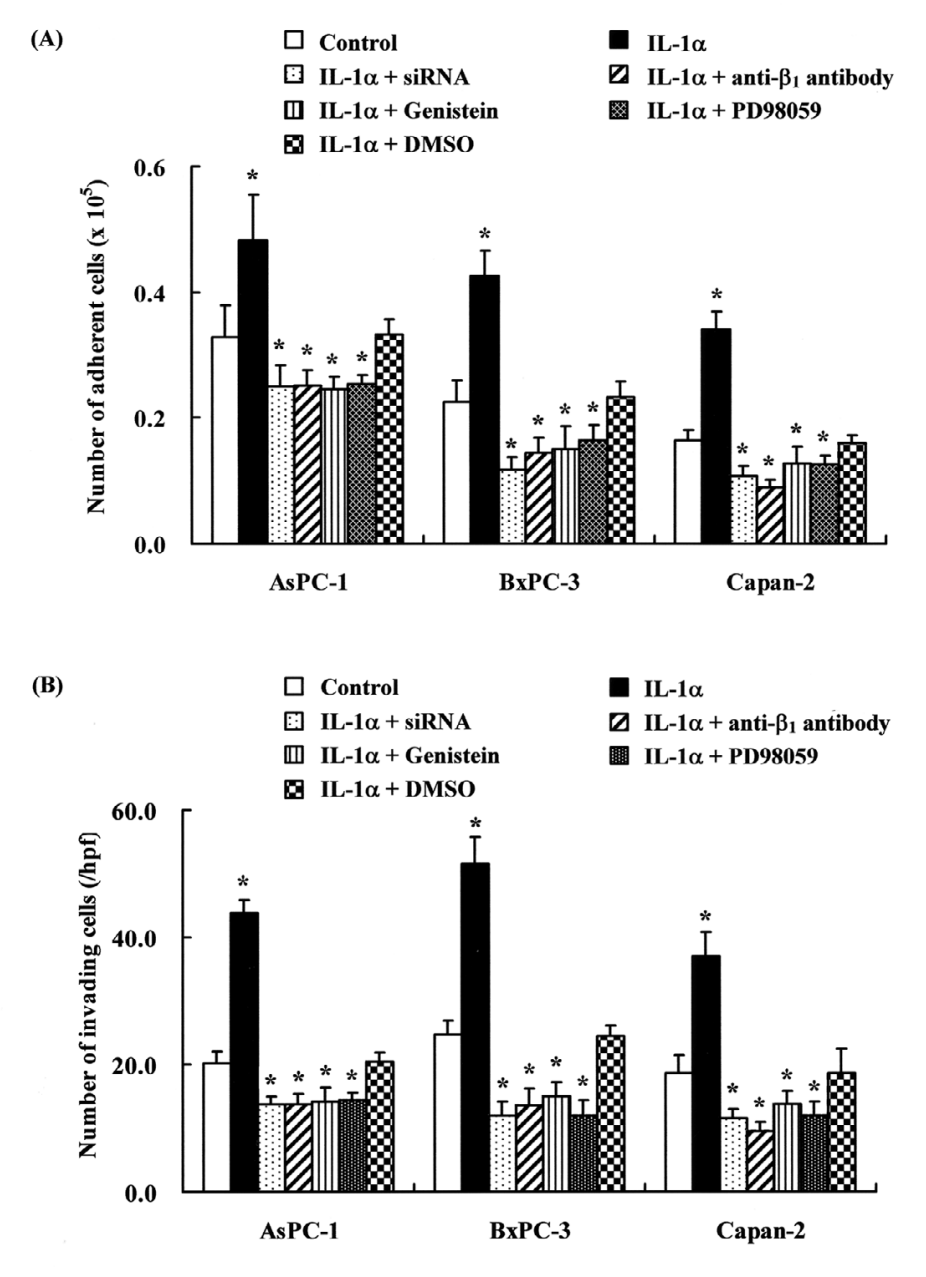
Involvement of FAK phosphorylation and integrin signaling with adhesion and invasion of pancreatic cancer cells. **(A) **AsPC-1, BxPC-3, and Capan-2 cells were incubated with 10 ng/ml IL-1α, with 10 ng/ml IL-1α and 60 μM Genistein, with 10 ng/ml rIL-1α and 25 μM PD98059, or with 10 ng/ml rIL-1α and equivalent amounts of DMSO vehicle for 24 h. FAK siRNA transfected cells were cultured with 10 ng/ml IL-1α for 24 h. After incubating for 30 min with/without anti-β_1 _antibody, cell adhesion assay was performed at 37°C for 30 min. Statistical significance was tested by one-way analysis of variance and post hoc test (Turkey Kramer multiple comparisons). The *p-*values indicate statistical significance between data in controls and each treatment. Bars indicate the s.d.*: *p *< 0.05. **(B) **After incubation for 30 min with/without antibody against β_1 _integrin, AsPC-1, BxPC-3, and Capan-2 cells were cultured with 10 ng/ml IL-1α, with 10 ng/ml IL-1α and 60 μM Genistein, with 10 ng/ml rIL-1α and 25 μM PD98059, or with 10 ng/ml rIL-1α and equivalent amounts of DMSO vehicle for 24 h in the inner chamber coated with Coll IV. FAK siRNA transfected cells were cultured with 10 ng/ml IL-1α for 24 h in the inner chamber. Statistical significance was tested by one-way analysis of variance and post hoc test (Turkey Kramer multiple comparisons). The *p*-values indicate statistical significance between data in controls and each treatment. Bars indicate the s.d. *: *p *< 0.05.

We investigated whether the inhibition of FAK had any effects on adhesive and invasive response in pancreatic cancer cells. Knockdown of FAK expression with siRNA inhibited IL-1α-induced adhesion and invasion (Fig. 4A, 4B). The inhibitory antibodies against β_1 _integrin subunit treatment similarly inhibited the IL-1α-induced enhancement of adhesion and invasion in all three pancreatic cancer cells. Genistein and PD98059 (a MEK inhibitor) also inhibited these enhancements of adhesion and invasion by IL-1α stimulation in pancreatic cancer cells. While not statistical affecting cellular apoptosis, the basal adhesive and invasive capabilities of these cells were also suppressed by siRNA, anti-β_1 _integrin antibody, Genistein, and PD98059 (Fig. 4A, 4B). DMSO vehicle had no effect on adhesion and invasion assays. These data suggest that FAK regulation and β_1 _integrin subunit may have critical roles in adhesive and invasive capabilities of pancreatic cancer cells.

### Activation of Ras and ERK pathway after IL-1α stimulation and Coll IV adhesion

**Figure 5:**
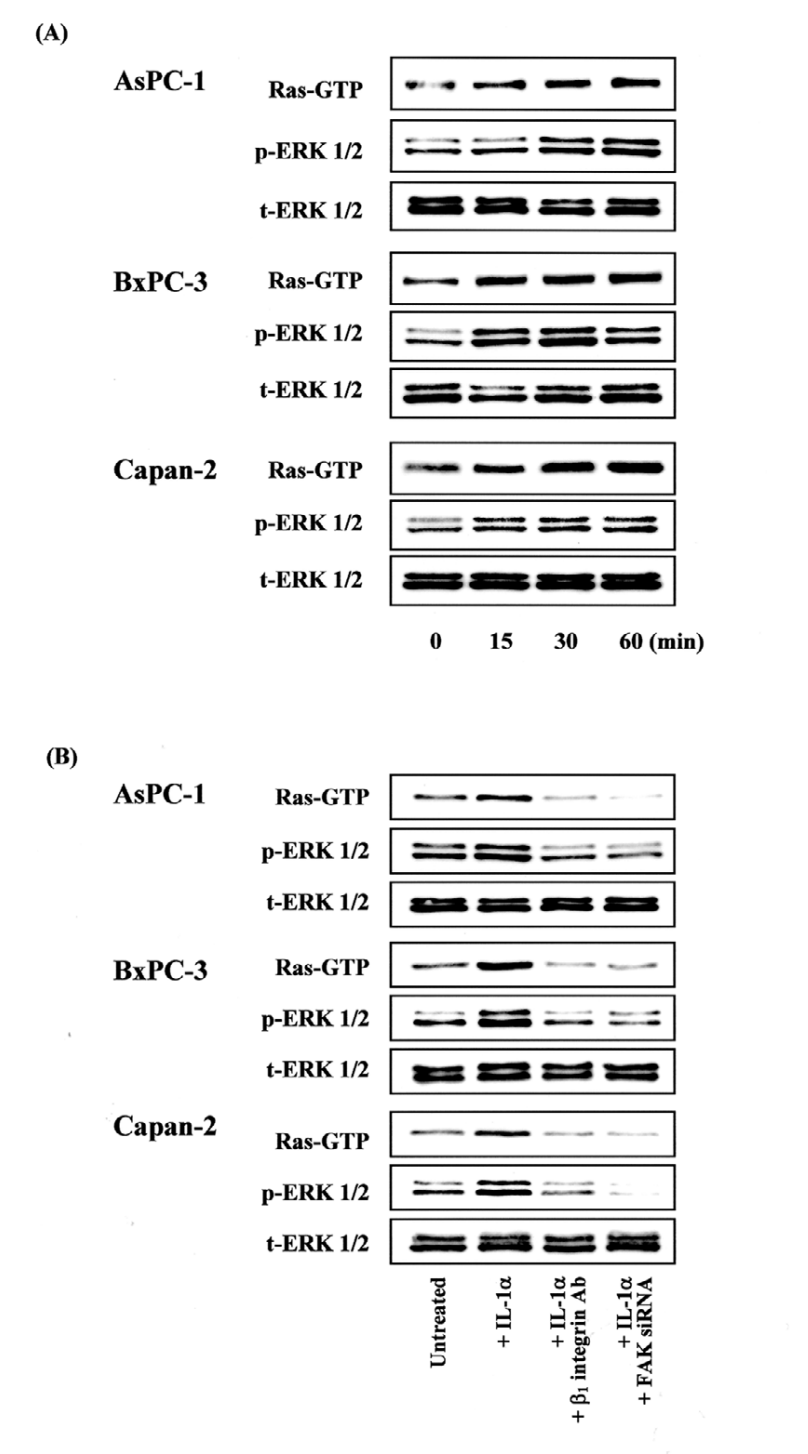
Involvement of FAK and β_1 _integrin subunit with the activation of Ras and ERK pathway in pancreatic cancer cells. Examination of Ras and downstream ERK activation was performed as described in Materials and Methods. Cell lysates were prepared according to the instructions provided in the Ras Activation Assay Kit, and affinity precipitation of GTP-bound Ras was performed using GST-tagged Raf-RBD. Levels of pull-downed Ras (Ras-GTP) were determined by anti-Ras immunoblotting.**(A) **AsPC-1, BxPC-3, and Capan-2 cells were serum starved for 24 h and then attached to Coll IV with 10 ng/ml IL-1α for 15, 30, or 60 min. The time-dependent Ras activation and ERK1/2 phosphorylation by adhesion to Coll IV was demonstrated. Detection of total ERK 1/2 levels served as a loading control.**(B) **AsPC-1, BxPC-3, and Capan-2 cells were serum starved for 24 h and then attached to Coll IV with 10 ng/ml IL-1α in the presence or absence of inhibitory antibodies against β_1 _integrin subunit for 30 min. FAK siRNA transfected pancreatic cancer cells were attached to Coll IV with 10 ng/ml IL-1α. Effects of IL-1α, FAK gene silencing, and β_1 _integrin blocking on the activation of Ras/ERK signaling pathway in pancreatic cancer cells were demonstrated. Detection of total ERK 1/2 levels served as a loading control.

We examined the activation of Ras/ERK pathway, a downstream target of FAK in pancreatic cancer cells, following adhesion of cells to Coll IV for 15, 30, or 60 min. IL-1α stimulation and Coll IV adhesion enhanced the activation of Ras, as evidenced by the increased Ras-GTP levels in three pancreatic cancer cell lines. Activation of Ras correlated with the phosphorylation of ERK. In contrast, IL-1α stimulation and Coll IV adhesion did not induce the Akt phosphorylation (data not shown). These results indicate that IL-1α and Coll IV adhesion may induce activation of ERK through a Ras-dependent pathway as a downstream of FAK activation (Fig. 5A). To evaluate whether FAK and β_1 _integrin affect IL-1α-induced activation of Ras and ERK, pancreatic cancer cells were transfected with FAK siRNA or treated with anti-β_1 _integrin antibody for 30 min before being exposed to rIL-1α for 30 min on Coll IV. Inhibition FAK expression and β_1 _integrin function inhibited the activation of Ras and phosphorylation of ERK in three pancreatic cancer cell lines (Fig. 5B). These results suggest that expression of FAK and β_1_-integrin has an important role in regulating IL-1α-induced activation of signaling pathways. Detection of total ERK 1/2 levels served as a loading control.

## Discussion

In this report, we demonstrate that FAK plays a critical role in adhesive behavior of pancreatic cancer cells via activating the Ras/ERK signaling pathways. FAK protein association with β_1 _integrin was increased when cells were attached on Coll IV and further increase was observed by stimulating with IL-1α. Knockdown of FAK expression with siRNA inhibited IL-1α-induced Ras/ERK activation with subsequent inhibition IL-1α-induced adhesion and invasion of pancreatic cancer cells on Coll IV while not statistical affecting cellular apoptosis.

The activation of FAK following cell adhesion to Coll IV and integrins engagement has been reported for many different cell types [3,4,7,25-27]. Recent reports have strongly implicated the FAK phosphorylation of lung cancer cells in adhesion to Coll IV [7]. In this study, the phosphorylation of FAK in pancreatic cancer cells was detectable with Coll IV adhesion in time-dependent manner. Furthermore, stronger FAK phosphorylation was observed by stimulation with IL-1α of pancreatic cancer cells adhered to Coll IV. The tyrosine phosphorylation of FAK was suppressed by Genistein, which has been reported as a potent inhibitor of several tyrosine kinases. It can be suggested that Genistein inhibits the activities of protein tyrosine kinases (PTKs), which are active in the upstream of FAK signaling pathway. Considering the effect of Genistein on FAK phosphorylation, it can be suggested that the induction of FAK phosphorylation in pancreatic cancer cells may proceed primarily through the inhibition of a specific protein tyrosine phosphatase. For instance, a dual-specific phosphatase, PTEN, has been shown to be associated with FAK and capable of FAK dephosphorylation [28-30]. It would be interesting to determine whether PTEN is associated with FAK in cancer cells after adhesion to Coll IV.

There is accumulating evidence that supports an important role of ERK pathway in promoting cell proliferation and invasion of cancer cell [4,31-35]. It has been reported in many cell lines that integrin dependent activation of MAPK requires FAK [24,36,37]. Coll IV-dependent activation of ERK1/2 in intestinal epithelial cells also requires FAK [3]. MAPK has been reported to regulate cell migration through the enhancement of myosin light chain phosphorylation [38]. Thus, the requirement for FAK activity in the efficient activation of MAPK could also be related to the effects of FAK on cell motility [39,40]. In addition, previous studies have indicated that loss of integrin signaling, such as FAK gene silencing and blocking of integrin signaling, can induce cellular apoptosis[14,20,41-44]. In contrast, these suppress of integrin signaling is reported to decrease the invasive behavior but not cellular apoptosis and proliferation in some cancer cells[2,45]. In this study, the similarity in the time course over which FAK and ERK1/2 were activated in response to IL-1α and adhesion to Coll IV of pancreatic cancer cells were observed. Both of knockdown of FAK expression with siRNA and blocking of β_1 _integrin inhibited IL-1α-induced Ras/ERK activation with subsequent inhibition IL-1α-induced adhesion and invasion of pancreatic cancer cells on Coll IV while not statistical affecting cellular apoptosis. Furthermore, we demonstrated that a MEK inhibitor PD98059 inhibited IL-1α-induced enhancement of adhesion and invasion in pancreatic cancer cells. Our results reveal that the relationship between FAK phosphorylation and the activation of its downstream Ras/ERK signaling pathway has an important role in the adhesive and invasive capabilities of pancreatic cancer cells. It has also been reported that IL-1 plays an important role in tumor invasion and angiogenesis [46-48]. Furthermore, recent reports demonstrated the involvement of IL-1 with the ERK1/2 signaling pathway activation [49-51]. Our results in this work are supported with these reports.

Integrin mediated signaling to ERK1/2 is dependent on the integrity of the actin cytoskeleton [52,53]. The disruption of cytoskeleton integrity completely inhibited fibronectin stimulated FAK tyrosine phosphorylation and ERK1/2 signaling. Also, in some cancer cells, the integrity of the cytoskeleton structure is required for ECM generated signals to ERK1/2. β_1 _integrin is reported to play an important role in adhesion and invasion of cancer cells [17-19]. And several intracellular signals have been suggested to mediate effects of IL-1, including activation of MAPK. Activation of MAPK by IL-1 subsequently induces the activator protein-1 and nuclear factor-κB DNA-binding activity, which promotes expression of the genes involved in cell survival, proliferation, and angiogenesis [54,55]. We also previously reported that the enhancement of α_6_β_1_-integrin expression by IL-1α acting through IL-1RI plays a critical role in adhesive and invasive behaviors in pancreatic cancer, and proved that the strong expression of the α_6 _integrin subunit in pancreatic cancer tissue significantly correlated with the poor prognosis and the presence of hepatic metastases in pancreatic cancer patients [15,16]. In this study, we investigated whether β_1 _integrin is physically associated with FAK or the activation of β_1 _integrin mediated signaling is sufficient to activate FAK in pancreatic cancer cells. We demonstrated that the enhancement of adhesion and invasion to Coll IV in pancreatic cancer cells is dependent on the presence of β_1 _integrin. Furthermore, we proved that FAK phosphorylation correlated with the activation of its downstream Ras/ERK signaling pathway. Our data indicates that the Coll IV-induced phosphorylation of FAK correlated with physical association of FAK with β_1 _integrin with subsequent the activation of ERK1/2 signaling pathways in pancreatic cancer cells.

## Conclusion

We demonstrated that IL-1α stimulation and cell adhesion to Coll IV enhanced the FAK protein association with β_1 _integrin and FAK phosphorylation. And these enhancements correlated with the activation of Ras/ERK signaling pathways in pancreatic cancer cells. IL-1α-induced activation of these signaling pathways can be inhibited by knockdown of FAK expression with siRNA, consistent with the inhibition of adhesive and invasive capabilities of pancreatic cancer cells. Based on these results, we suggest that the modification of IL-1, FAK, and integrins functions might be a novel therapeutic approach to aggressive spread of pancreatic cancer.

## Methods

### Cell culture

The human pancreatic cancer cell lines, AsPC-1, BxPC-3 and Capan-2, were provided from the American Type Culture Collection (Rockville, MD, USA). The AsPC-1 and BxPC-3 cells were maintained in RPMI 1640 (Gibco BRL, Eggenstein, Germany) supplemented with 10 % fetal calf serum (FCS). Capan-2 cells were maintained in Dulbecco modified Eagle medium (Gibco BRL) with high glucose and 10 % fetal calf serum. All cells were incubated at 37°C in a humidified atmosphere of 5 % CO_2 _in air.

### Reagents and Antibodies

rIL-1α was provided by Diaclone (Besançon, France). Coll IV (human) and Genistein were purchased from SIGMA (Saint Louis, MO, USA). PD98059 was purchased from New England Biolabs Inc (Beverly, MA, USA). The monoclonal antibodies used included anti-β1 (P5D2) from Chemicon International Inc. (Temecula, CA, USA); anti-phosphotyrosine (4G10) and anti-FAK (4.47) from Upstate Biotechnology Inc. (Lake Placid, NY, USA). The polyclonal anti-phospho-Akt (587F11), anti-phospho-ERK 1/2 and anti-ERK 1/2 antibodies were from Cell Signaling Technology (Beverly, MA, USA).

### Western blot analysis

Cells were lysed in lysis buffer (50 mM Tris-HCl, pH 7.5, 150 mM NaCl, 1 mM CaCl_2_, 1 % Triton X-100, 0.1 % SDS, 0.1 % Nonidet P-40, 2 mM PMSF, 1 mM vanadate, 5 μg/ml Trasylol, 10 μM Pepstatin A and 10 μM leupeptin). Protein concentrations were determined with a BCA protein assay kit (Pierce, Rockford, IL, USA). The amounts of samples were 50 μg per each lane. Lysates were separated by 10 % SDS-polyacrylamide gel electrophoresis (SDS-PAGE), transferred to polyvinylidene difluoride membranes (Immobilon PVDF; Nihon Millipore Ltd., Tokyo, Japan) and immunoblotted with each antibody. β-actin Western blots were served as controls.

### Immunoprecipitation

Pancreatic cancer cells lysates were centrifuged at 14,000 rpm at 4°C for 15 min and the supernatants (750 μg of protein) were used for immunoprecipitation with either anti-β1 integrin (P5D2) or anti-FAK (4.47) antibody at 4°C overnight. After incubation for 1 h with antibody, either protein A- or protein G-sepharose was added to the lysates and incubated overnight at 4°C. The bead bound complexes were pelleted, washed several times with lysis buffer in PBS and boiled with SDS sample buffer for 5 min before loading on 10 % SDS-PAGE. For Western blot analysis, the proteins were transferred to polyvinylidene difluoride membranes after SDS-PAGE Specific binding was detected with the enhanced chemiluminescence system (ECL; Amersham Life Science Ltd., Buckinghamshire, United Kingdom).

### RNA interference (siRNA)

Pancreatic cancer cells were transfected with siRNA for FAK and with control nonspecific siRNA using FAK siRNA/siAb™ Assay Kits (Upstate Biotechnology Inc.) according to the manufacture's instruction. Briefly, cells were grown in 35 mm dishes and overlaid with the transfection mixture containing siRNA at a concentration of 200 pmol/well. After 4 h incubation, complete medium with 10 % FCS was added and cells were incubated for another 48 h.

#### TUNEL assay

Detection of apoptosis was performed by TUNEL assay. After incubating for 24 h with/without anti-β_1 _integrin antibody, pancreatic cancer cells were collected by centrifugation, fixed in 4 % paraformaldehyde (pH 7.4) and then stained and analyzed for apoptosis using an In Situ Cell Death Detection Kit, Fluorescein (Roche Diagnostics GmbH, Penzburg, Germany). Fixed cells were permeabilized using a mixture containing terminal deoxynucleotidyltransferase and fluorescein-dUTP at 37°C for 60 min. Flow cytometric analysis using a FACS scan (Becton Dickinson Immunocytometry Systems, Mountain View, CA, USA) was done to quantitate apoptosis[56].

### Cell adhesion assay

Adhesion assay was performed as described previously with some modifications [15]. 24-well plates were coated either with Coll IV (5.0 μg/cm^2^) or 3 % bovine serum albumin (BSA) in phosphate-buffered saline (PBS). Briefly, after incubating for 24 h with/without rIL-1α (10 ng/ml), Genistein (60 μM), PD98059 (25 μM), or equivalent amounts of DMSO vehicle, cells were added (2 × 10^5 ^cells/well) to each well and incubated at 37°C and 5 % CO_2 _for 15, 30, or 60 min. After removing unattached cells, the number of adherent cells was counted directly by light microscopy. Before the stimulating experiments with IL-1α were attempted, a concentration of 10 ng/ml was determined to be the lowest effective concentration for stimulating experiments (data not shown). In some experiments, 0.5 μg/ml anti-β_1 _integrin antibodies were added to cells for 30 min prior to seed to the well. Before the blocking experiments were attempted, a concentration of 0.5 μg/ml was determined to be the lowest effective concentration for blocking experiments (data not shown). Experiments were performed in triplicate and repeated at least three times.

### Cell invasion Assay

In vitro invasion assays were performed as described previously with some modifications [15]. Polycarbonate filters (6.3-mm diameter, 8- μm pore size) of cell culture inserts (BD Biosciences Discovery Labware, Franklin Lakes, NJ, USA) were coated with 5.0 μg/cm^2 ^Coll IV. Cancer cells were added (1 × 10^5 ^cells/well) to the inner chamber of a cell culture insert and incubated at 37°C for 24 h, either with 10 ng/ml rIL-1α, with 10 ng/ml rIL-1α and 60 μM Genistein, with 10 ng/ml rIL-1α and 0.5 μg/ml of anti-β_1 _integrin antibody, with 10 ng/ml rIL-1α and 25 μM PD98059, or with 10 ng/ml rIL-1α and equivalent amounts of DMSO vehicle. To quantitate invasion, the filters were fixed in 70 % ethanol for 30 min and stained with Giemsa. Cells were removed from the upper surface of the filters by rubbing gently with a cotton-tipped applicator. Cells that had invaded through the membrane were counted in five random microscope fields of the lower filter surface.

### Ras activation assay

Ras activation state was determined using the Ras Activation Assay Kit provided from Upstate. Briefly, after serum starved for 24 h, pancreatic cancer cells were incubated on the 35-mm well coated with Coll IV (5.0 μg/cm^2^) in serum-free medium with/without rIL-1α (10 ng/ml) for 15, 30, or 60 min. Then, cells were harvested and lysed in lysis buffer (100 mM HEPES, pH 7.5, 200 mM NaCl, 1 % Nonidet P-40, 10 mM MgCl_2_, 5 mM EDTA and 10 % glycerol), and supernatant prepared by centrifugation for 5 min at 4°C at 14,000 *g*. Ras-GTP from various treated lysates was "pulled down" using the GST fusion protein corresponding to human Ras binding domain of Raf-1 bound to agarose. The presence of Ras-GTP was detected by Western blotting using anti-Ras antibody (Upstate). In some experiments, 0.5 μg/ml anti-β_1 _integrin antibodies were added to cells for 30 min prior to seed to the well.

### Statistical analysis

Statistical comparisons were made using the Student's *t *test for paired observations or by one-way ANOVA with a post hoc test for multiple comparisons. Statistical significance was indicated by *p *< 0.05. Data are presented as mean ± standard deviation (s.d.). Each experiment was repeated 3 times and was carried out in triplicate.

## Abbreviations

ECM, extracellular matrix; FAK, focal adhesion kinase; Coll IV, collagen type IV; IL, interleukin; IL-1RI, IL-1 receptor type I; ERK, extracellular signal-regulated kinase; MAPK, mitogen activated protein kinase; PI3-K, phosphatidylinositol 3-kinase; TUNEL, deoxynucleotidyl transferase-mediated nick end labeling; PTK, protein tyrosine kinase; FCS, fetal calf serum; SDS-PAGE, SDS-polyacrylamide gel electrophoresis; BSA, bovine serum albumin; PBS, phosphate-buffered saline.

## Authors' contributions

HS carried out the Western blots, immunoprecipitations, and the investigation of Ras activity in addition to the drafting of the manuscript. YO and HF contribute the adhesion and invasion assays and statistical analyses. YM and TH performed the cell culture, adhesion assay, and the literature search. HT designed the experiments and contributed to the writing of the manuscript. TM conceived the project and aided in experimental design. All authors read and approved the final manuscript.
